# Differences in the Concentration of the Fecal Neurotransmitters GABA and Glutamate Are Associated with Microbial Composition among Healthy Human Subjects

**DOI:** 10.3390/microorganisms9020378

**Published:** 2021-02-13

**Authors:** Hend Altaib, Kohei Nakamura, Mayuko Abe, Yassien Badr, Emiko Yanase, Izumi Nomura, Tohru Suzuki

**Affiliations:** 1 The United Graduate School of Agricultural Science, Gifu University, 1-1 Yanagido, Gifu 501-1193, Japan; x6103007@edu.gifu-u.ac.jp (H.A.); knak2007@gifu-u.ac.jp (K.N.); e-yanase@gifu-u.ac.jp (E.Y.); 2 Graduate School of Natural Science and Technology, Gifu University, Gifu 501-1193, Japan; y4521002@edu.gifu-u.ac.jp; 3 Faculty of Applied Biological Sciences, Gifu University, 1-1 Yanagido, Gifu 501-1193, Japan; yaseen.badr@vetmed.dmu.edu.eg (Y.B.); i_nomura@gifu-u.ac.jp (I.N.); 4 Department of Animal Medicine, Faculty of Veterinary Medicine, Damanhour University, El-Beheira 22511, Egypt

**Keywords:** gut microbiota, *Bifidobacterium*, gut–brain axis, neurotransmitters, GABA

## Abstract

Recent studies have shown that the gut microbiota modulates the physical and psychological functions of the host through several modes of action. One of them is mediating the production of active neurotransmitters, such as serotonin and gamma-aminobutyric acid (GABA). GABA is the major inhibitory neurotransmitter in the central nervous system. Here, we analyzed the relationship between fecal GABA concentration and microbial composition in more than 70 human participants. The gut microbiome composition was analyzed using next-generation sequencing based on 16S ribosomal RNA. High-performance liquid chromatography was used to evaluate the neurotransmitters GABA and glutamate. The GABA level was detected in a broad range (0–330 µg/g feces). The participants’ samples were classified into high (>100 µg/g), medium (10–100 µg/g), and low (<10 µg/g) groups, based on fecal GABA concentration. The results reveal that the microbiome of the high-GABA samples had lower alpha diversity than the other samples. Beta diversity analysis showed significant (*p* < 0.05) separation between the high-GABA samples and others. Furthermore, we surveyed the abundance of specific GABA producer biomarkers among the microbiomes of tested samples. The family Bifidobacteriaceae exhibited high abundance in the microbiome of the high-GABA group. This study demonstrated that *Bifidobacterium* abundance was associated with high fecal GABA content in healthy human subjects. These results may aid the development of potential probiotics to improve microbial GABA production, which can support the maintenance of the physical and psychiatric health of the host.

## 1. Introduction

The gut microbiota comprises several microorganisms, including bacteria, archaea, and fungi, which inhabit the gastrointestinal tract (GIT) of mammals. It was reported that the number of bacterial cells in the human body is 3.8 × 10^13^, on average, which is almost equal to the number of adult human body cells [1,2]. The GIT bacteria constitute a considerable percent of the total bacteria residing in the human body [1]. The gut microbiota is often called the “forgotten organ” owing to its broad spectrum of health benefits for the host [3]. The gut microbiota acts as a key modulator of host digestion, metabolism, and immune response. Recent research has shown that the effect can extend beyond the gastrointestinal tract to affect the mental health of the host through bidirectional communication between the gut and brain, which is referred to as the microbiota–gut–brain axis [4,5]. Signals transfer between the gut and the brain via neural, endocrine, immune, and humoral links [6]. One important link is the neural pathway in which the gut microbiota mediates the production of active neurotransmitters that pass from the gut to its target organs, including the brain.

Gamma-aminobutyric acid (GABA) is the most abundant inhibitory neurotransmitter in the central nervous system (CNS) [7]. It is biosynthesized by glutamate decarboxylase (EC 4.1.1.15), which catalyzes the α-decarboxylation of glutamate to GABA and CO_2_. Glutamate is an excitatory neurotransmitter in the CNS [8]. Hence, the balance between these two neurotransmitters is crucially important to maintain a stable nervous condition. GABA production has been reported in several species belonging to the families Bifidobacteriaceae, Lactobacillaceae, Bacteroidaceae, Enterococcaceae, Propionibacteriaceae, and Streptococcaceae [9,10]. *Bifidobacterium* exhibits the ability to produce GABA from specific strains belonging to the species *B. dentium*, *B. angulatum*, *B. adolescentis*, and *B. longum* subsp. *infantis* [11,12]. GABA-producing bacteria are considered glutamate consumers as glutamate activates enzymatic conversion using microbial glutamate decarboxylases.

Microbial GABA can pass from the gut to other organs through several pathways, including the blood or vagal pathways [13]. It has been reported that mental disorders, such as depression, are negatively correlated with the abundance of GABA-producing Bacteroides [14]. Further, accumulating evidence from animal trials suggests that the ingestion of GABA-producing bacteria supports relief from psychiatric illnesses, such as depression, and physical ailments, such as diabetes [15,16,17]. As the majority of available evidence for GABA’s relation to microbial composition has been performed in animals, there is a need for more evidence from human cohorts to encourage the application of these microbes as probiotic agents.

Understanding the relationship between microbial composition and the level of fecal neurotransmitters GABA and glutamate can highlight the vital role of some microbes which can redirect the microbiome activity towards GABA or glutamate production. In this study, we aimed to assess microbial diversity among human subjects with different fecal GABA and glutamate levels.

## 2. Materials and Methods

### 2.1. Study Subjects

From March 2020 to August 2020, stool samples were obtained from 77 participants. Eligible participants were randomly selected from those who did not receive antibiotic treatment, GABA-containing food, or GABA medication at least three months before sample collection. This information was obtained by a personal questionnaire for each participant. Participants who did not match these criteria were excluded from the study. Additionally, none of the participants were an athlete or involved in any intensive physical activity. Participants were from different geographical origins. Their ages ranged from 1 month to 80 years. All were apparently healthy with no systemic or psychiatric illnesses.

### 2.2. Ethical Statement

All experimental protocols were approved by the Institutional Ethics Review Board of Gifu University (certificate number: 2019–283), approved on 3 March 2020. Written informed consent was obtained from each participant.

### 2.3. Fecal Sample Manipulations

Stool samples were collected in sterile 12 mL tubes with tight caps. Samples were frozen immediately at −20 °C and delivered to the laboratory using cool containers. Thereafter, samples were stored at −80 °C, directly after being obtained, until used for amino acid quantification and DNA extraction. All processing was performed within five days after receiving samples.

### 2.4. High-Performance Liquid Chromatography (HPLC)

The GABA and glutamate concentrations were determined by HPLC using pre-column fluorescent derivatization with the *o*-phthalaldehyde (OPA) method as previously described [18]. Briefly, fecal samples were diluted 10 times with pure water (*w*/*v*), homogenized, and the liquid fraction filtered through a 0.45-µm membrane filter. Two solutions were prepared on the same day of HPLC analysis. OPA solution (a) was prepared by dissolving 5 mg OPA (Wako, Osaka, Japan) powder in 1.5 mL 100% ethanol and 3.5 mL borate buffer (0.1 mol/L). An amount of 10 µL 3-mercaptoprobionic acid (Wako) was dissolved in 100 mL of borate buffer (0.1 mol/L) to make solution (b). Amounts of 300 µL of solution (a) and 600 µL of solution (b) were added to 100 µL of filtrated sample and incubated for 2 min at room temperature. The derivatized product was promptly analyzed using HPLC (Agilent Technologies, Waldbronn, Germany) with an ODS column (Cosmosil 5C_18_-MS-II, 3.0ID × 150 mm), followed by fluorescence detection (Ex 350 nm Em 450 nm). The mobile phase was composed of reagents A (20 mM KH_2_PO_4_ (pH 6.9), H_3_PO_4_) and B (CH_3_CN/CH_3_OH/H_2_O 45/40/15, *v*/*v*/*v*). Compounds were eluted using a gradient program: 0–9 min, 100% A; 9–12 min, 89% A; 12–21 min, 78% A, with a flow rate of 0.7 mL/min at 35 °C. HPLC-grade CH_3_CN and CH_3_OH were purchased from Wako. GABA (Wako) and glutamate (Sigma, Louis, MO, USA) were used for standard curve preparation.

### 2.5. DNA Manipulation and Next-Generation Sequencing (NGS)

Genomic DNA was extracted from fecal samples using an ISOFECAL kit for Beads Beating (Nippon Gene, Tokyo, Japan). The polymerase chain reaction (PCR) was performed with the barcoded primers, Fw (5′ GTGCCAGCMGCCGCGGTAA 3′) and Rv (5′ GGACTACHVGGGTWTCTAAT 3′), targeting the V3-V4 region of the bacterial 16S ribosomal RNA gene [19]. It produced a fragment length of approximately 550 base pairs (bp). The PCR was performed using 2× KAPA HiFi HotStart ReadyMix (Kapa Biosystems, Woburn, MA, USA) according to the manufacturer’s instructions. Subsequently, the PCR amplicons were purified using AgencourtR AMPureR XP beads (Beckman Coulter, Beverly, MA, USA). Dual indices and Illumina sequencing adapters were attached using Nextera XT (Illumina, San Diego, CA, USA) in the index PCR step. The concentration of PCR amplicons was measured using a Qubit^®^ Fluorometer (Thermo Fisher Scientific, Waltham, MA, USA). Quality control for the created library was performed using a Bioanalyzer (Agilent Technologies, Santa Clara, CA, USA). Pooled libraries were denatured with NaOH, diluted with hybridization buffer, and subsequently heat-denatured prior to MiSeq sequencing. PhiX DNA was used as an internal control in each run. The NGS of amplicons was carried out on Illumina MiSeq (Illumina) using the MiSeq Reagent Kit v3, following which 300-bp paired-end reads were produced.

### 2.6. Bioinformatics and Statistical Analysis Tools

Preprocessing of sequences obtained by NGS and extraction of operational taxonomic units (OTUs) was performed using the software *mothur* (version 1.41.0) [20]. OTUs of amplicons were designated at 97% sequence similarity. Taxonomic assignments were performed with *mothur*, based on non-redundant SILVA datasets (release 132) [21]. A phylogenetic tree for the *phyloseq* object was calculated using the *clearcut* function implemented in *mothur* [22]. The chimera sequences were removed by the Chimera.vsearch (https://github.com/torognes/vsearch; accessed on 8 October 2020) function implemented in *mothur* [23]. OTUs that occurred only once (singletons) or twice (doubletons) among all samples were removed. Next, the number of reads of all samples was rarefied to be equal in size at the minimum read within samples (approximately 33,000 reads per sample) using the *phyloseq* package of R software.

Alpha diversity of samples was measured using Shannon, observed, and Chao1 indices. Calculation of alpha diversity indices and production of their diagrams were performed with the plot_richness function implemented in the *phylosq* package. Non-metric multidimensional scaling (NMDS), an unconstrained and distance-based ordination method, was performed with Bray–Curtis dissimilarity matrices and produced using the *phyloseq* and vegan packages of R software [24,25,26]. Differences in the microbial community structure, calculated using Bray–Curtis distances, were analyzed statistically using permutational multivariate analysis of variance with distance matrices (PERMANOVA) using the ADONIS command implemented in the vegan package. OTUs designated at the family level of classification were used for heatmap and cluster analyses. Bray–Curtis dissimilarity distance was applied to these analyses. A heatmap combined with a dendrogram was generated using the gplot [27] and cluster [28] packages of R. Distance-based redundancy analysis (db-RDA) was performed with the Bray–Curtis distance matrix of the family-level taxonomy of OTUs using the vegan package of R. Species scores of abundant taxa (top 10) were also displayed on db-RDA plots. Linear discriminant analysis (LDA) effect size (LEfSe) [29] was performed using the microbiomeMarker package of R under default settings except for the LDA cutoff, which was set to 4 in this study [30]. The results of LEfSe were further analyzed with the “test_multiple_groups” function implemented in the microbiomeMarker package of R. Statistical analysis was performed using the ANOVA test followed by a post hoc test (Tukey–Kramer test) to assess the biological relevance of the obtained results. The correlation coefficient between GABA and glutamate concentrations was estimated statistically with the cor.test function in the stats package (https://www.rdocumentation.org/packages/stats/versions/3.6.2/topics/cor.test; accessed on 3 February 2021).

## 3. Results

### 3.1. Analysis of Fecal GABA and Glutamate Levels from 77 Participants

**Figure 1:**
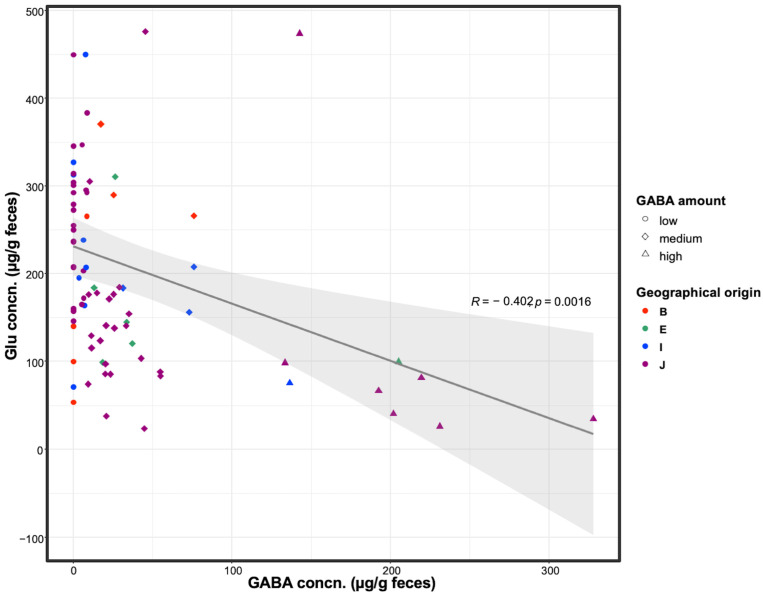
Fecal GABA and glutamate concentrations. Fecal GABA and glutamate contents were analyzed among healthy human participants. Participants were divided based on GABA productivity into high, medium, and low groups. Circle, diamond, and triangle symbols represent participants categorized as low-, medium-, and high-GABA productivity groups, respectively. Different colors represent the geographical origin of each participant: B, South Asia; E, Northeast Africa; I, Southeast Asia; J, East Asia. Regression curve is displayed on the figure with a deep gray line, showing a negative correlation between fecal GABA and glutamate concentrations. Confidence interval (95%) is expressed in a light gray color. The correlation coefficient (R) and *p*-value of the regression curve are also shown on the plot.

To investigate the microbiome activity for GABA production, fecal GABA and glutamate levels were evaluated in 77 participants. These participants were from different geographical origins, including Northeast Africa, Southeast Asia, South Asia, and East Asia. There were 55 participants from Japan and 22 from other geographical areas. The GABA and glutamate levels were detected in a broad range (0–330 µg/g feces and 55–475 µg/g feces, respectively). The correlation coefficient between GABA and glutamate concentrations was estimated as −0.402 with a 95 % confidence interval [−0.596, −0.162], which indicates a negative co-relation between both neurotransmitters. Participants’ samples were divided into high, medium, and low, based on their fecal GABA content. The high, medium, and low groups were defined as those with productivity (µg-GABA/g-feces) ≥ 100, 10–100, and <10, respectively (Figure 1). Notably, the high-GABA group samples had low glutamate content and vice versa, indicating that the microbiome was actively involved in converting the available glutamate in the gut to GABA. Sample data are summarized in Table S1.

### 3.2. Reduced Alpha Diversity of the High-GABA Group

The quality check and preprocessing of NGS data yielded a total of 1885 OTUs with a median of 171 OTUs per sample. To unify the depth of all sample data, singletons or doubletons were first removed and all samples were rarefied at an even depth (*n* = 24,770). The total coverage of each sample ranged from 99.5 to 99.9%. The number of reads for all samples was rarefied to be equal to the minimum read within samples. Rarefaction plots, grouped by GABA level and geographical origin, are displayed in Figure S1A,B.

**Figure 2:**
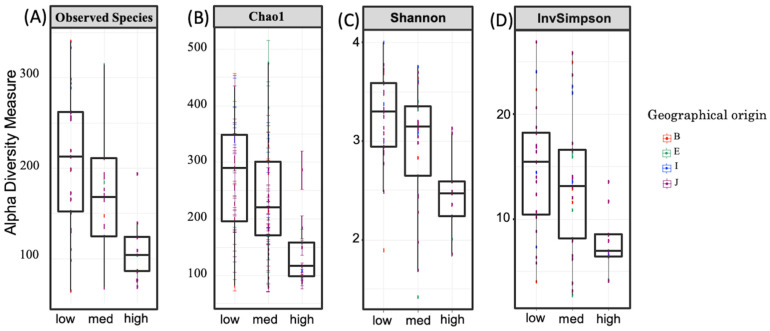
Alpha diversity among fecal GABA groups. Alpha diversity, measured by observed species (**A**) and Chao1 (**B**), Shannon diversity (**C**), and inverse Simpson indices (**D**), is plotted for examined samples, i.e., in high-, medium-, and low-GABA groups. Box plots depict microbiome diversity and abundance differences according to each test. The horizontal line inside the box represents the median. Outliers and individual sample values are represented by dots. Different colors represent the geographical origin of each participant: B, South Asia; E, Northeast Africa; I, Southeast Asia; J, East Asia. All alpha diversity measurements shown here significantly decreased in the high-GABA group compared to those in the low group.

Alpha diversity was subsequently quantified by the total number of observed species, Chao1 richness, the Shannon diversity index, which estimates both OTU richness and evenness, and inverse Simpson, which estimates evenness. Figure 2 shows the alpha diversity measurements for the high-GABA group versus the medium and low groups. The alpha diversity of the high (H)-GABA group compared to the medium (M) and low (L) groups was significantly reduced in observed species (H–L; P_Observed_ = 0.0006), Shannon diversity (H–L; P_Shannon_ = 0.001) (H–M; P_Shannon_ = 0.04), and Chao1 richness (H–L; P_Chao1_ = 0.002) (M–H; P_Chao1_ = 0.03). Statistical comparison between the M and L groups showed no difference in Shannon diversity (M–L; P_Shannon_ = 0.21), Chao1 richness (M–L; P_Chao1_ = 0.39), and observed species (M–L; P_Observed_ = 0.07). A summary of alpha diversity indices is shown in Table S2.

### 3.3. Microbial Composition Differed between GABA Groups

**Figure 3:**
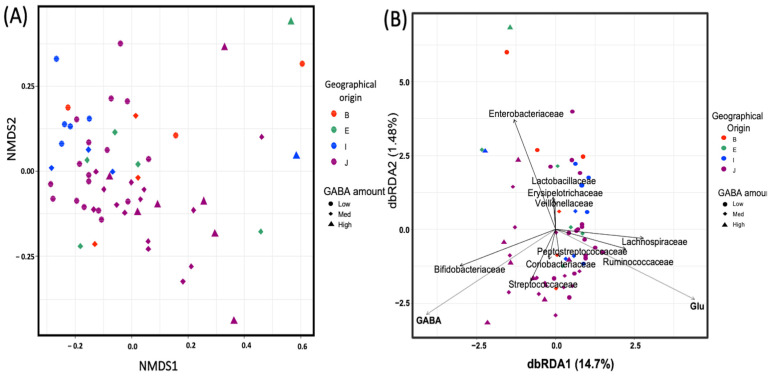
Beta diversity and community similarity analysis among fecal gamma-aminobutyric acid (GABA) groups. (**A**) Non-metric multidimensional scaling (NMDS) plot based on the distance matrix of operational taxonomic units (OTUs) designated at the family level of taxonomic classification calculated using the Bray–Curtis model. (**B**) Distance-based redundancy analysis (db-RDA) using the Bray–Curtis dissimilarity matrix calculated with OTUs designated at the family level of taxonomy. The environmental variables were statistically significant (*p* < 0.01), and the top 10 most abundant taxa are displayed. Circle, diamond, and triangle symbols represent participants categorized as low-, medium-, and high-GABA productivity groups, respectively. Different colors represent the geographical origin of each participant: B, South Asia; E, Northeast Africa; I, Southeast Asia; J, East Asia.

As a distance-based discrimination approach, NMDS ordination was applied to investigate dissimilarities in the microbial composition between tested samples based on GABA production. The NMDS plot for community structure based on the Bray–Curtis matrix of the family-level taxonomy is displayed in Figure 3A. The microbiome of individuals with high GABA content showed a shift to the right, which indicates compositional differences. This was confirmed by the ADONIS test, which provided more precise information regarding the homogeneity of the dispersion between the two sample groups. Significant differences (*p* < 0.05) were observed between groups (Table 1).

Relationships among microbial community structures and environmental variables were examined with db-RDA based on the Bray–Curtis distance matrix of the family-level taxonomy of OTUs. This revealed that the abundance of the families Bifidobacteriaceae and Streptococaceae was closely associated with GABA levels, while that of the families Lachnospiraceae and Ruminococcaceae was closely associated with glutamate levels (Figure 3B).

### 3.4. Trend toward Clustering of the Microbiome of Individuals with High GABA Content

**Figure 4:**
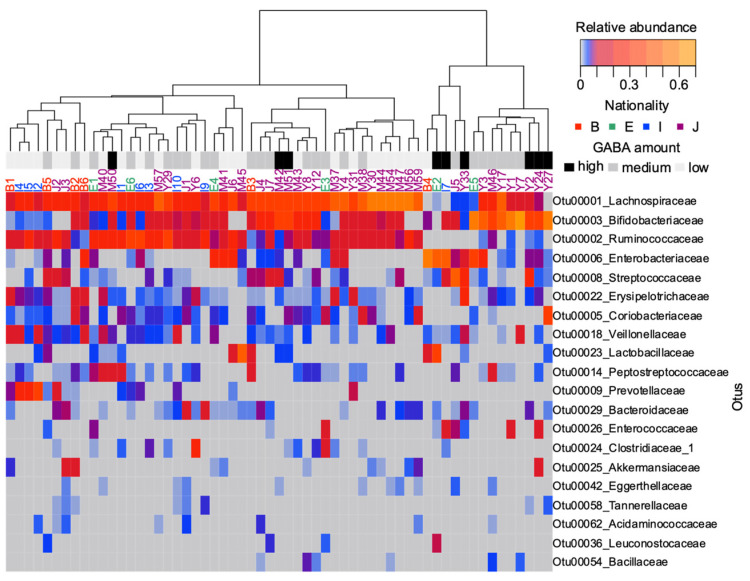
Heatmap of the top 20 abundant taxa in the examined human fecal samples. Operational taxonomic units (OTUs) were collapsed at the family-level taxonomy. A dendrogram was constructed with the beta flexible method based on the distance matrix of OTUs, calculated using the Bray–Curtis model. The geographical origin of each participant is represented by different colors: B, South Asia; E, Northeast Africa; I, Southeast Asia; J, East Asia.

We subsequently investigated whether fecal GABA content could reflect a notable difference in the microbial composition of individuals. The top 20 abundant taxa are displayed as a heatmap in Figure 4. A distant matrix computed with all OTUs was used to produce the dendrogram. Hierarchical cluster analysis revealed that the community structure profiles of most high-fecal GABA samples were separated from other analyzed microbiomes (Figure 4). Six out of nine high-GABA samples were clustered in the same group. All nine samples showed an abundance of Bifidobacteriaceae, except for one participant, ID: E2. This participant had a unique abundance of two other GABA producer candidates, Lactobacillaceae and Leuconostocaceae. One participant, ID: Y33, showed a relatively low abundance of Bifidobacteriaceae. Nonetheless, other GABA producer candidates were detectable in this microbiome, Y33, including Streptococcaceae, Bacteroidaceae, and Enterococcaceae. Ruminococcaceae exhibited a relatively high abundance in the low and medium groups. Lachnospiraceae was abundant in most analyzed samples.

### 3.5. Bacterial Taxonomic Differences between GABA Groups (Specific OTUs)

**Figure 5:**
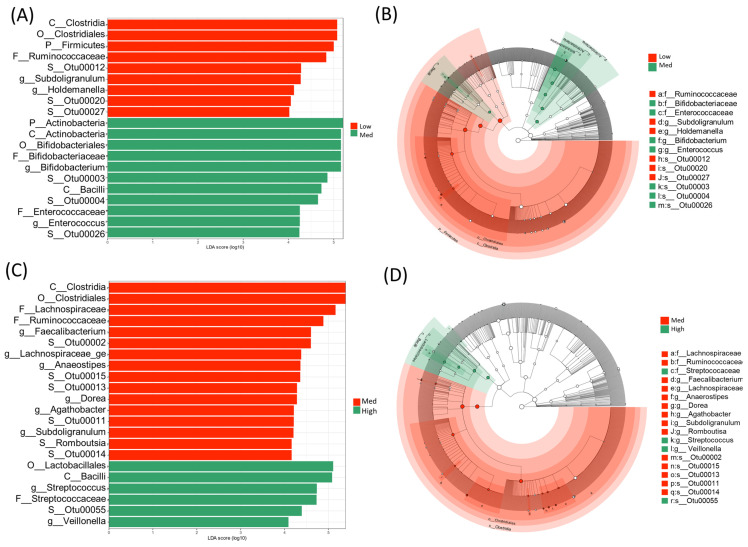
Linear discriminate analysis (LDA) effect size (LEfSe) showing the characteristics of the microbial community composition between the low- and medium-gamma-aminobutyric acid (GABA) groups in panels (**A**,**B**) as well as between the medium- and high-GABA groups in panels (**C**,**D**). (**A**,**C**) LEfSe (LDA scores 10^4^ and more) displaying statistical and differentially abundant taxa in each group. (**B**,**D**) Cladogram showing the microbiome differences at different phylogenetic levels. The central point represents the root of the tree (bacteria) and each ring displays the next (lower) taxonomic rank (p__, phylum; c__, class; o__ order; f__, family; g__, genus). The diameter of each circle represents the relative abundance of each taxon.

**Figure 6:**
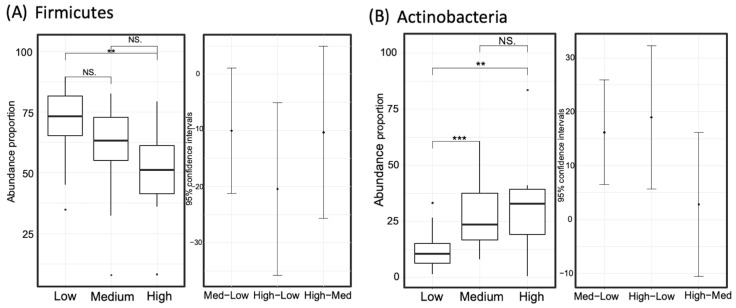
Comparison of the relative taxon abundance of the phyla Firmicutes (panel **A**) and Actinobacteria (panel **B**) among the three gamma-aminobutyric acid (GABA) groups (high, medium, and low). Significance codes: <0.001 ***, <0.01 **, <0.1 “NS”.

To identify biomarkers from the high-GABA group, we used LEfSe to comprehensively and accurately investigate compositional differences between the three GABA groups. Specific OTUs that showed the strongest effect for group differentiation were identified between the two sample groups (Figure 5). At the phylum level, Firmicutes was dominant in the microbiome of the low-GABA group (Figure 6A), whereas the phylum Actinobacteria was highly dominant in the microbiome of the high and medium groups (Figure 6B). The microbiomes of the low and medium groups were characterized by the dominance of the class Clostridia, which was primarily represented by the families Ruminococcaceae and Lachnospiraceae. In contrast, the microbiome of the high-GABA group was characterized by a high abundance of the class Actinobacteria and orders Bifidobacteriales and Lactobacillales, which were principally represented by the families Bifidobacteriaceae, Enterococcaceae, and Streptococcaceae (Figure 5).

**Figure 7:**
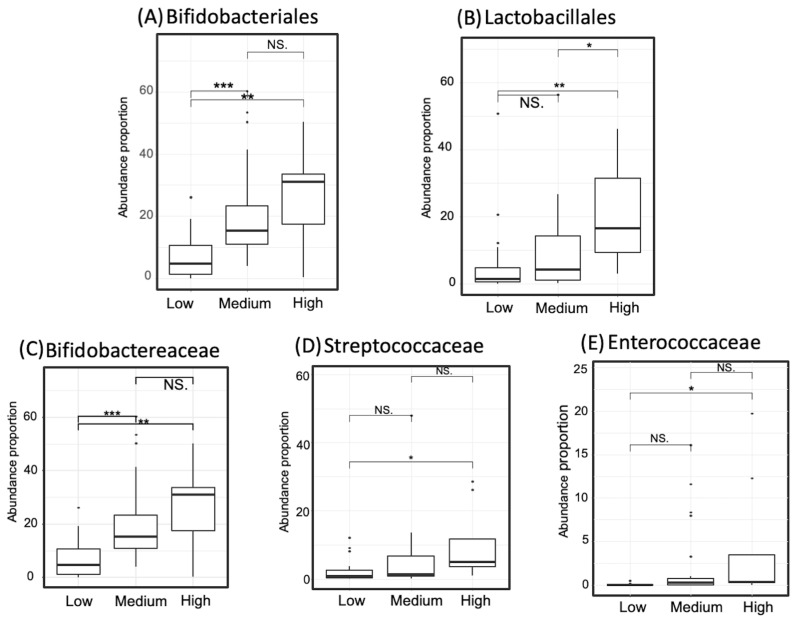
Proportion of the taxa that occurred abundantly in the high-gamma-aminobutyric acid (GABA) group. Two taxa at the order level, comprising Bifidobacteriales (Panel **A**) and Lactobacillales (Panel **B**), are displayed. Three taxa at the family level are demonstrated incorporating Bifidobacteraceae (Panel **C**), Streptococcaceae (Panel **D**), and Enterococcaceae (Panel **E**). Significance codes: <0.001 ***, <0.01 **, <0.05 *, <0.1 “NS”.

**Figure 8:**
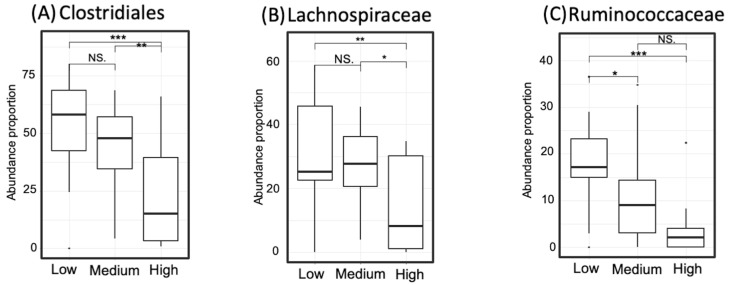
Proportion of taxa that occurred abundantly in the low-gamma-aminobutyric acid (GABA) group including Clostridiales (Panel **A**), Lachnospiraceae (Panel **B**), and Ruminococcaceae (Panel **C**). Significance codes: <0.001 ***, <0.01 **, <0.05 *, <0.1 “NS”.

The abundance of taxa that showed dominance in the high- or low-GABA group was compared between the three GABA groups. The degree of significance among the groups is displayed in Figure 7 and Figure 8. The order Bifidobacteriales and family Bifidobacteriaceae were more abundant in the high- and medium-GABA groups than the low group. A significant (*p* < 0.01) difference was noticed between the low and high groups, and a larger difference (*p* < 0.001) was detected between the low and medium groups, while no significant difference was found between the medium and high groups (*p* < 0.1). The order Lactobacillales was more abundant in the high-GABA group than other groups. Both the families Streptococcaceae and Enterococcaceae were more abundant in the high-GABA group than in the low-GABA group, as shown in Figure 7. The order Clostridiales and the two families Lachnospiraceae and Ruminococcaceae were dominant in the low-GABA group (Figure 8).

To identify the microbiomes of individuals causing these differences, the abundance of the major taxa, associated with high or low GABA levels, was presented using a colored NMDS plot, as shown in Figure S2. The abundance of the dominant taxa in the high- and low-GABA groups is represented by gradient colors, which enables the clear visualization of the taxa distribution among tested samples.

## 4. Discussion

Gut bacteria have the ability to produce numerous bioactive compounds including neurotransmitters, immune stimulants, and essential vitamins [31,32]. Several studies emphasized the contribution of gut microbiota-derived materials in the maintenance of host physical and psychological conditions [16,17]. Assessing the neuroactive potential and composition of the human gut microbiota can suggest the crucial role of some microbes [33,34]. In the current study, we analyzed the relationship between microbial composition and the levels of the fecal neurotransmitters, GABA and glutamate. We found promising biomarker bacteria associated with high fecal GABA concentrations. Fecal GABA and glutamate concentrations showed substantial remodeling of the gut microbiota at different levels.

In this study, we observed a negative correlation between fecal GABA and glutamate concentrations. This finding suggests that approximately 400 µg of glutamate pooled in the colon, presumably produced by the gut microbiota. Then, a part of the glutamate pool was converted to GABA by the GABA-producing bacteria. Hence, the fecal GABA and glutamate level diverted between participants. GABA is biosynthesized by irreversible α-decarboxylation of glutamate via the action of glutamate decarboxylase [35]. In vitro studies revealed the ability of some bacteria to produce GABA [9]. To discover this ability in vivo, we examined the microbial composition and diversity of participants with different fecal GABA and glutamate levels.

Low microbial alpha diversity was associated with high fecal GABA levels compared to other samples of low and medium GABA concentrations. Consistent with that, rarefication of raw NGS data showed a relative decrease in the number of species in the high-GABA group. The highly diverse microbiome is thought to be advantageous for maintenance of the microbial ecosystem. Reduction in alpha diversity was reported to have an association with unhealthy conditions and specific diseases [36]. Nonetheless, the high abundance of beneficial bacteria such as bifidobacteria in cases of low alpha diversity is often a step forward to restore the microbial diversity [37]. Still, more research is needed to reveal the desired level of microbial diversity for maintenance of host health. Assuming a positive relationship, the reduced alpha diversity found in the high-GABA group might reflect a bacterial community associated with GABA production, which was verified using db-RDA and LEfSe analysis. Further, the ADONIS test confirmed the differences in homogeneity between GABA groups.

db-RDA shifts the focus onto specific taxa associated with high GABA and glutamate levels. The families Bifidobacteriaceae and Streptococcaceae were associated with high GABA production. The families Ruminococaceae and Lacnospiraceae were associated with high glutamate levels. Notably, several species of Bifidobacteriaceae and Streptococcaceae have been reported to be high-GABA producers [11,12,38]. Analysis of the top 15 taxa showed abundance of Bifidobacteriaceae in all high-GABA samples except one sample (ID: E5), which revealed a unique abundance of Leuconostocaceae and Lactobacillaceae (presented in a heatmap). Interestingly, several species belonging to these families were reported as GABA producers [9], indicating the contribution of GABA-producing bacteria to the detected GABA level in our study.

The LEfSe results confirm the relatively high abundance of the family Bifidobacteriaceae in the high-GABA group. The (OTU0003) showed higher abundance in the medium-GABA group compared to the low group. Basic Local Alignment Search Tool analysis of this OTU revealed that it belonged to the *B. adolescentis* species. A recent study has shown that this species is a key member of the gut microbiota involved in GABA production [11]. Such capability is proposed to alter the classification of *Bifidobacterium* from ordinary probiotic bacteria to potential psychobiotic bacteria. The term psychobiotics was first introduced by Dinan and colleagues to describe mind-altering germs [4]. It was subsequently broadened to include any exogenous influence whose effect on the brain is bacterially mediated [39].

These results indicate the important role of bifidobacteria in improving GABA production in the gut. Previous studies have shown that the gut microbiota affects the levels of excitatory and inhibitory neurotransmitters, such as serotonin, GABA, and dopamine [40]. Consistent with these previous findings, in the present study, we found a positive association between the abundance of the GABA producer families, Bifidobacteriaceae, Enterococcaceae, and Streptococcaceae, and fecal GABA concentrations. Compared to other GABA producers in the gut, *Bifidobacterium* and *Streptococcus* have a simple GABA production system composed of two genes, *gadB*, which encodes glutamate decarboxylase, and *gadC*, which encodes a glutamate–GABA anti-porter [12,38,41]. Other more complex systems exist in other GABA producers, such as *lactobacillus* and *Enterococcus* [42,43].

As GABA is an inhibitory neurotransmitter and glutamate is an excitatory one [13,44], the existence of bacteria that can decarboxylate glutamate to GABA can produce a new therapeutic agent for relieving stress, improving sleep quality, and also supporting psychiatric illnesses, particularly in cases related to an imbalance between glutamate and GABA levels. Analysis of both neurotransmitters is important for understanding the microbiome activity for the conversion of glutamate to GABA. Our study reveals that the gut microbiota seems to play a crucial role for balancing GABA–glutamate levels, where GABA-producing bacteria were positively associated with high GABA levels and negatively associated with high glutamate levels. Previous studies showed that balancing between both neurotransmitters, GABA and glutamate, was linked to several psychiatric disorders such as autism [45], multiple sclerosis [46], and neuro-Bechet’s disease [47]. In autism patients, an altered fecal concentration of GABA and glutamate was observed, where high fecal levels of glutamate were detected in children with autism and low fecal GABA was detected in other subtypes of autism [48]. The probiotic formulation with abilities to consume high levels of glutamate and convert them to GABA promises to aid in the development of new supportive therapy for autism and other related psychiatric disorders. The current study suggests that GABA producer bacteria, bifidobacteria, are a good candidate in this field. Nonetheless, our study was limited to healthy participants, and thus future studies will be warranted to include both healthy and diseased subjects.

## 5. Conclusions

The current study reveals that microbial diversity and composition differ based on GABA concentration. This suggests the important role of some commensal gut microbes in mediating GABA production and glutamate consumption. This study also highlights the importance of assessing the neuroactive potential and composition of the gut microbiota, which emphasize the imperative role performed by certain microbes for the production or consumption of specific neurotransmitters, such as GABA-producing bifidobacteria proposed in our study. The finding of this study may aid the development of potential probiotics to improve microbial GABA production, which can support the maintenance of the mental conditions and psychiatric health of the host.

## Figures and Tables

**Table 1:** ADONIS test between each of the two sample groups.

Group	F. Model	R2	*p*-Value	*p*-Adjust
Low vs. Medium	5	0.09	0.001	0.003
Low vs. High	8	0.19	0.001	0.003
Medium vs. High	3	0.09	0.004	0.012

## Supplementary Materials

The following are available online at https://www.mdpi.com/2076-2607/9/2/378/s1, Figure S1: Rarefaction curves for analyzed samples, Figure S2: Non-metric multi-dimensional scaling (NMDS) of community structure for all tested samples, Table S1: Summary of human volunteers’ data, Table S2: Alpha diversity between GABA groups.
