# Supplementary material for: Differences in the Concentration of the Fecal Neurotransmitters GABA and Glutamate Are Associated with Microbial Composition among Healthy Human Subjects
Source: Microorganisms. 2021 Feb 13;9(2):378. doi: 10.3390/microorganisms9020378 (PMC7918917; doi:10.3390/microorganisms9020378)
Supplement: Supplementary file 1 [file microorganisms-09-00378-s001.zip › microorganisms-1090172-supplementary.docx]

Table S1. Summary of human volunteers’ data

| **Serial number** | **ID** | **GABA ug/gram** | **Glutamate ug/gram** | **Geographical origin** | **Age** | **Gender** |
| --- | --- | --- | --- | --- | --- | --- |
| **1** | I1 | 7.6 | 450.2 | Southeast Asia | 26 years | F |
| **2** | I2 | 3.5 | 195.4 | Southeast Asia | 26 years | M |
| **3** | I3 | 31.3 | 183.6 | Southeast Asia | 11months | F |
| **4** | I4 | 6.2 | 238.4 | Southeast Asia | 12 years | F |
| **5** | I5 | ND | 312.6 | Southeast Asia | 10 years | F |
| **6** | I6 | ND | 327.3 | Southeast Asia | 28 years | F |
| **7** | I7 | 136.37 | 75.8 | Southeast Asia | 27 years | M |
| **8** | I8 | ND | 70.3 | Southeast Asia | 4 months | F |
| **9** | I9 | 7.3 | 156.3 | Southeast Asia | 27 years | F |
| **10** | I10 | 7.6 | 207.8 | Southeast Asia | 23 years | F |
| **11** | B1 | ND | 100.0 | South Asia | 28 years | M |
| **12** | B2 | 7.6 | 265.9 | South Asia | 29 years | M |
| **13** | B3 | 17.2 | 370.8 | South Asia | 29 years | F |
| **14** | B4 | ND | 140.2 | South Asia | 1 month | F |
| **15** | B5 | 25.2 | 289.8 | South Asia | 32 years | M |
| **16** | B6 | ND | 53.8 | South Asia | 36 years | M |
| **17** | E1 | 13.0 | 184.0 | Northeast Africa | 37 years | F |
| **18** | E2 | 205.2 | 99.8 | Northeast Africa | 12 months | F |
| **19** | E3 | 18.37 | 99.0 | Northeast Africa | 37 years | M |
| **20** | E4 | 37.2 | 120.6 | Northeast Africa | 2.5 years | M |
| **21** | E5 | 33.6 | 144.9 | Northeast Africa | 8 months | F |
| **22** | E6 | 26.4 | 310.6 | Northeast Africa | 25 years | F |
| **23** | J1 | 25.2 | 176.6 | East Asia | 55 years | F |
| **24** | J2 | ND | 207.2 | East Asia | 76 years | F |
| **25** | J3 | ND | 158.3 | East Asia | 46 years | F |
| **26** | J4 | 11.1 | 129.3 | East Asia | 21 years | F |
| **27** | J5 | 33.2 | 140.9 | East Asia | 23 years | F |
| **28** | J6 | ND | 237.9 | East Asia | 48 years | F |
| **29** | J7 | 14.7 | 178.0 | East Asia | 22 years | F |
| **30** | Y1 | 20.2 | 97.4 | East Asia | 41 years | M |
| **31** | Y2 | 142.5 | 474.1 | East Asia | 10 years | M |
| **32** | Y3 | 44.7 | 23.6 | East Asia | 41 years | F |
| **33** | Y4 | 23.2 | 85.8 | East Asia | 15 years | M |
| **34** | Y6 | ND | 272.4 | East Asia | 68 years | F |
| **35** | Y7 | 20.0 | 86.0 | East Asia | 71 years | M |
| **36** | Y8 | 10.2 | 305.2 | East Asia | 9 years | M |
| **37** | Y10 | 19.4 | 44.5 | East Asia | 40 years | F |
| **38** | Y12 | 35.0 | 154.5 | East Asia | 66 years | F |
| **39** | Y13 | 10.7 | 115.3 | East Asia | 12 years | M |
| **40** | Y14 | 10.5 | 174.8 | East Asia | 13 years | M |
| **41** | Y15 | 5.7 | 157.9 | East Asia | 32 years | F |
| **42** | Y16 | 6.5 | 201.9 | East Asia | 34 years | M |
| **43** | Y17 | 16.9 | 123.8 | East Asia | 54 years | F |
| **44** | Y24 | 219.5 | 81.7 | East Asia | 1 years | M |
| **45** | Y26 | 9.2 | 166.9 | East Asia | 27 years | M |
| **46** | Y27 | 328.0 | 34.7 | East Asia | 8 months | F |
| **47** | Y29 | 45.3 | 476.2 | East Asia | 3 years | F |
| **48** | Y30 | ND | 304.2 | East Asia | 33 years | M |
| **49** | Y31 | ND | 236.6 | East Asia | 28 years | M |
| **50** | Y32 | 19.8 | 253.7 | East Asia | 32 years | F |
| **51** | Y33 | 231.0 | 26.1 | East Asia | 1 year | M |
| **52** | Y34 | 52.8 | 82.6 | East Asia | 37 years | F |
| **53** | M36 | 80.5 | 339.4 | East Asia | 73 years | F |
| **54** | M37 | 5.4 | 347.1 | East Asia | 83 years | M |
| **55** | M38 | 25.8 | 138.1 | East Asia | 47 years | F |
| **56** | M39 | ND | 279.8 | East Asia | 47 years | F |
| **57** | M40 | ND | 313.9 | East Asia | 47 years | F |
| **58** | M41 | ND | 157.3 | East Asia | 79 years | F |
| **59** | M42 | 192.4 | 66.9 | East Asia | 30 years | F |
| **60** | M43 | ND | 208.2 | East Asia | 30 years | F |
| **61** | M44 | ND | 293.5 | East Asia | 49 years | F |
| **62** | M45 | ND | 145.9 | East Asia | 78 years | F |
| **63** | M46 | 28.9 | 184.8 | East Asia | 52 years | F |
| **64** | M47 | ND | 254.1 | East Asia | 24 years | F |
| **65** | M48 | ND | 248.0 | East Asia | 46 years | F |
| **66** | M49 | 53.3 | 88.2 | East Asia | 47 years | F |
| **67** | M50 | 201.8 | 40.8 | East Asia | 9 years | M |
| **68** | M51 | 133.5 | 98.64 | East Asia | 15 years | M |
| **69** | M52 | 29.9 | 134.7 | East Asia | 20 years | F |
| **70** | M53 | 11.3 | 75.98 | East Asia | 53 years | M |
| **71** | M54 | ND | 449.8 | East Asia | 46 years | F |
| **72** | M55 | 42.4 | 102.3 | East Asia | 49 years | M |
| **73** | M56 | ND | 345.76 | East Asia | 49 years | F |
| **74** | M57 | ND | 160.8 | East Asia | 26 years | M |
| **75** | M58 | 23.1 | 172.7 | East Asia | 21 years | F |
| **76** | M59 | 8.5 | 383.8 | East Asia | 76 years | F |
| **77** | M60 | 6.7 | 291.1 | East Asia | 23 years | F |

Values are the average of duplicate or triplet analysis of each sample

Table S2. Alpha diversity between GABA groups.

|  |  |  | 95% confidence level | |  |
| --- | --- | --- | --- | --- | --- |
| Model | Compared groups | diff | lwr | upr | adjusted p-value |
| Invsimpson | High_Low | -6.9 | -13 | -1 | 0.01 |
|  | Medium_Low | -1.2 | -5 | 3 | 0.76 |
|  | Medium_High | 5.7 | 0.2 | 11 | 0.04 |
| Shannon | High_Low | -0.8 | -1 | -0.3 | 0.001 |
|  | Medium_Low | -0.3 | -0.6 | 0.1 | 0.210 |
|  | Medium_High | 0.5 | 0.003 | 1 | 0.048 |
| ACE | High_Low | -128 | -218 | -37 | 0.003 |
|  | Medium_Low | -35 | -101 | 31 | 0.4 |
|  | Medium_High | 93 | 2 | 184 | 0.04 |
| Chao1 | High_Low | -130 | -219 | -41 | 0.002 |
|  | Medium_Low | -35 | -100 | 29 | 0.4 |
|  | Medium_High | 94 | 6 | 183 | 0.03 |
| Observed | High_Low | -103 | -166 | -40 | 0.0006 |
|  | Medium_Low | -43 | -88 | 3 | 0.07 |
|  | Medium_High | 60 | -2 | 123 | 0.06 |
